# Ranking’s Abstract and Braithwaite’s Retrospect

**Published:** 1850-05

**Authors:** 


### ARTICLE VII.

Ranking's Abstract of the Medical Sciences, and Braithwaite's Retrospect continue to make their regular semi-annual visits to our table, and continue to receive a hearty welcome, as valuable repertories of the improvements in Medical Science. They are republished in this country; the former by Lindsay & Blakiston of Philadelphia; the latter by Daniel Adee, New York. Terms $1,50 per annum in advance.

